# Professional and Personal Quarrels

**Published:** 1870-04

**Authors:** 


### Professional and Personal Quarrels.

In New York, Boston, Chicago and some other cities, members of the medical profession are fully engaged in professional and personal quarrels. Looking matters over from an outside standpoint we conclude that truly, "people who live in glass houses must not throw stones." Who of the combattants are going to have their windows shadowed or darkened does not yet appear, but really the whole profession is liable to be lowered rather than elevated; practices are exposed in physicians holding the most honorable and responsible public positions, which if shown in the conduct of the most unpretending private practitioner, would stamp him as charlatan and cheat. We have received various pamphlets setting forth in detail, both sides of pending controversies but have not space or inclination to give any detailed account of their contents. We desire to conceal and cover all the faults of the profession which we cannot correct.

